# Rapid and Ultrasensitive Detection of Mutations and Genes Relevant to Antimicrobial Resistance in Bacteria

**DOI:** 10.1002/gch2.202000066

**Published:** 2020-11-30

**Authors:** François Huber, Hans Peter Lang, Daniela Lang, Daniel Wüthrich, Vladimira Hinić, Christoph Gerber, Adrian Egli, Ernst Meyer

**Affiliations:** ^1^ Swiss Nanoscience Institute (SNI) Department of Physics University of Basel Klingelbergstrasse 82 Basel CH‐4056 Switzerland; ^2^ Clinical Bacteriology and Mycology, University Hospital Basel Applied Microbiology Research Department of Biomedicine University of Basel Petersgraben 4 Basel 4031 Switzerland

**Keywords:** bacterial antibiotic resistances, diagnostics, MRSA, nanosensors, single nucleotide polymorphisms

## Abstract

The worldwide emergence of multidrug‐resistant (MDR) bacteria is associated with significant morbidity, mortality, and healthcare costs. Rapid and accurate diagnostic methods to detect antibiotic resistance are critical for antibiotic stewardship and infection control measurements. Here a cantilever nanosensor‐based diagnostic assay is shown to detect single nucleotide polymorphisms (SNPs) and genes associated with antibiotic resistance in Gram negative (*Pseudomonas aeruginosa*) and positive (*Enterococcus faecium*) bacteria, representing frequent causes for MDR infections. Highly specific RNA capture probes for SNPs (*ampR^D135G^* or *ampR^G154R^*) or resistance genes (*vanA*, *vanB*, and *vanD*) allow to detect the binding of bacterial RNA within less than 5 min. Serial dilutions of bacterial RNA indicate an unprecedented sensitivity of 10 fg µL^−1^ total RNA corresponding to less than ten bacterial cells for SNPs and 1 fg µL^−1^ total RNA for *vanD* detection equivalent to single bacterial cell sensitivity.

## Graphical abstract

A new nanosensor array method is presented to investigate antibiotic resistances in bacteria. Single nucleotide polymorphisms in ampicillin resistance markers from gram negative *Pseudomonas aeruginosa* and vancomycin resistance genes in gram positive *Enterococcus faecium* are studied. The method shows unprecedented sensitivity and specificity (1 bacterial cell) as well as drastically reduced times to obtain a diagnosis, compared to standard procedures.

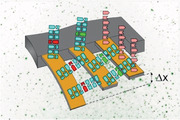

## 1 Introduction

Multidrug resistant (MDR) bacteria pose an important threat to human health and achievements of modern medicine. In a recent report^[^ ^1^ ^]^ (WHO, UN ad hoc interagency coordinating group on antimicrobial resistance), the World Health Organization estimates that bacteria with antibiotic resistance account for at least 700 000 deaths globally per year. A figure that could increase to ten million cases of deaths globally by the year 2050.^[^ ^2^ ^]^ There is a clear danger that in a post‐antibiotic era, common infections, such as pneumonia, blood stream infections, and sexually transmitted diseases, literally will become untreatable and surpass cancer‐related deaths.

Bacteria harbor a broad range of resistance mechanisms against antimicrobial substances, many of which are genetically encoded. Therefore, determination of genotypes can be transferred to phenotypic behavior during antibiotic treatment. Single nucleotide polymorphisms (SNPs) may alter antibiotic target binding sites or expression of, e.g., porins resulting in antibiotic drug resistance. Resistance can also be acquisition of mobile genetic elements such as plasmids and transposons, harboring genes encoding for modified target binding sites or enzymes able to cleave antibiotic substances.^[^ ^3^ ^]^ Bacteria with these resistance mechanisms have emerged worldwide due to a lack of antibiotic stewardship in veterinary and human medicine.^[^ ^4^ ^]^

**Figure 1:**
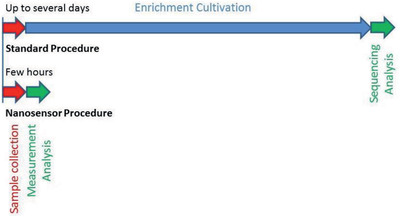
A schematic representation highlights the differences between the standard workflow and the nanosensor workflow. The enrichment and cultivation step can be omitted and shortens the procedure drastically from days to few hours. Particularly cultivation of fastidious or anaerobic bacteria, such as *M. tuberculosis* can take several weeks. Furthermore, the sample preparation step, extraction of DNA/RNA can be included into the sample collection stage.

In a clinical context, rapid and reliable detection of MDR bacteria is critical for treatment adaptations, antibiotic stewardship, and infection control. Current available technologies for screening and identification of MDR bacteria have been recently reviewed.^[^ ^5^ ^]^ A key problem of phenotypic resistance tests is the requirement to culture and subculture specimen in order to reach single colonies for subsequent processing on, e.g., broth dilutions. The process to determine antibiotic resistance breakpoints usually takes 48–72 h.^[^ ^6^ ^]^ Based on antimicrobial resistance (AMR) profiles, treatments can be tailored to more effective drugs, which is associated with patient mortality.^[^ ^7^ ^]^ However, slow growing bacterial species as well as fastidious or anaerobic bacteria pose challenges for rapid AMR testing (**Figure** **1**). Molecular diagnostic testing such as PCR or isothermal amplification^[^ ^8^ ^]^ may overcome these obstacles by rapid amplification of DNA and specific detection. Current molecular diagnostics to detect gene or SNP associated with antibiotic resistance are commonly used and allow rapid detection within a few hours. However, these technologies are often expensive and detection of resistance mechanisms directly from specimen may be challenging due to inhibitory substances and sensitivity issues.^[^ ^9^, ^10^ ^]^

A recently introduced method based on an atomic force microscopy (AFM) cantilever may overcome the previously mentioned obstacles.^[^ ^11^, ^12^, ^13^, ^14^, ^15^ ^]^ A variation of this technology measures the activity of bacteria in real time,^[^ ^16^, ^17^ ^]^ whereby the determination of low‐frequency fluctuations (<1 kHz) allows to distinguish living from dead bacteria. Antibiotic susceptibility can be inferred based on fluctuation changes while adding different concentrations of antibiotics. The method still requires single bacterial colonies from previous cultures. The method would require preselection of the bacteria and requires placement of bacteria onto a single cantilever only without specifically immobilizing them.

**Figure 2:**
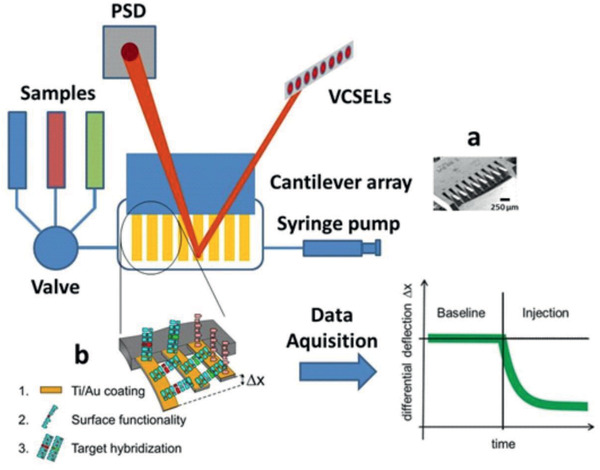
Microfabricated arrays (a) scanning electron microscopy picture of an array used in this study). Arrays of eight identical silicon cantilevers with 250 µm pitch, a length of 750 µm, a thickness of 500 nm were provided by the Micro‐ and Nanomechanics group at the IBM Zurich Research Laboratory and coated with a layer of 3 nm titanium and 20 nm gold. A cantilever array is functionalized using a Microdrop inkjet printer, incubated for 1 h at room temperature and then mounted in a microfluidic cell. At the same time total RNA can be extracted from bacterial samples and is then diluted in 0.03× SSC. Different concentrations between 1 fg µL^−1^ and 40 ng µL^−1^ of total RNA were injected sequentially at a flow rate of 5 µL min^−1^ for a total of 100 µL volume by pulling the liquid through the liquid cell with the help of a syringe pump and a multiway valve. All measurements were performed at 28 °C in a temperature controlled box. Time‐multiplexed vertical‐cavity surface‐emitting lasers (VCSEL) with regulated power supply (operated at 1 Hz, wavelength 760 nm, Avalon Photonics, Zurich, Switzerland) were used in combination with adjustable optics to yield a pitch of 250 µm. A 2D linear position‐sensitive detector, PSD (SiTek Electro Optics, Partille, Sweden) was used for beam deflection readout of each cantilever with an accuracy of 0.1 nm. The signals were preamplified, and the data were acquired using a National Instruments (Austin, TX) PCMCIA 16XE‐50 (16 bit, 200 kS s^−1^) data‐acquisition card. The instrument is driven by LABVIEW software to control liquid exchange via a syringe pump (GENIE, Kent Scientific, Torrington, CT), and a multiway valve selector (Rheodyne, Rohnert Park, CA) and data acquisition (b) binding of target molecules to the probes on the sensor surface). Data processing is done using Origin Software.

Here, we applied nanomechanical microcantilever arrays using a fundamentally different approach, providing internal references for differential readout to eliminate thermal drift and nonspecific binding (**Figure** **2**). Such devices represent ultra‐sensitive sensors for the detection of biochemical interactions in liquid environments.^[^ ^18^, ^19^ ^]^ Extracted RNA from isolates or samples are directly measured without pre‐concentration. Each nanosensor is coated with probes (gene specific oligonucleotide) on a sensitive layer for molecular recognition. The binding of the bacterial RNA sequence is mechanically transduced to the cantilever surface, resulting in bending of the cantilever. In this study, we first focus on the identification of SNPs in bacterial genes (*ampR^D135G^*, *ampR^G154R^*),^[^ ^20^ ^]^ that are involved in modulating gene expression and secondly on whole gene detection of typical resistance genes (*vanA*, *vanB*, *vanD* genes).

## 2 Results and Discussion

### 2.1 Detection of SNPs

First, we focus on single nucleotide polymorphisms in the bacterial genome. In order to assess the assay's sensitivity to detect SNPs in the bacterial genome, we have designed probes able to bind specific mutations. As a proof of concept, we have selected the *ampR* gene, an important transcriptional regulator^[^ ^21^ ^]^ in Gram negative bacteria, which is able to alter *ampC* gene expression. AmpC is a beta‐lactamase enzyme able to cleave many commonly used betalactam antibiotics. Two previously described and common mutations, i.e., *ampR^D135G^* and *ampR^G154R^* were used and compared to wild type *ampR^wt^*. These mutations are associated with phenotypic resistance against a series of beta‐lactam antibiotics. We used four different oligonucleotides as probes, 19 bases in length, where the location of the point mutation is in the center to increase hybridization specificity (**Table** **1**).

**Table 1:** Sequences of AmpR oligonucleotides used in this study: The different SNPs are highlighted by capitalized letters

Designation	Sequence	Position range	Position	*T*_m_ ^a)^
*ampR* ***^135wt^***	gcggcgatg **T** cgacgcggt	394–413	404	27.3 °C
*ampR* ***^D135G^***	gcggcgatg **C** cgacgcggt	“	“	28.0 °C
*ampR* ***^135ref^***	gcggcgatg **A** cgacgcggt	“	“	27.1 °C
*ampR* ***^154wt^***	cctcggtgc **C** gtgccaggc	452–470	460	28.1 °C
*ampR* ***^G154R^***	cctcggtgc **G** gtgccaggc	“	“	27.9 °C
*ampR* ***^154ref^***	cctcggtgc **A** gtgccaggc	“	“	27.1 °C

^a)^ *T*_m_ stands for melting temperature

**Figure 3:**
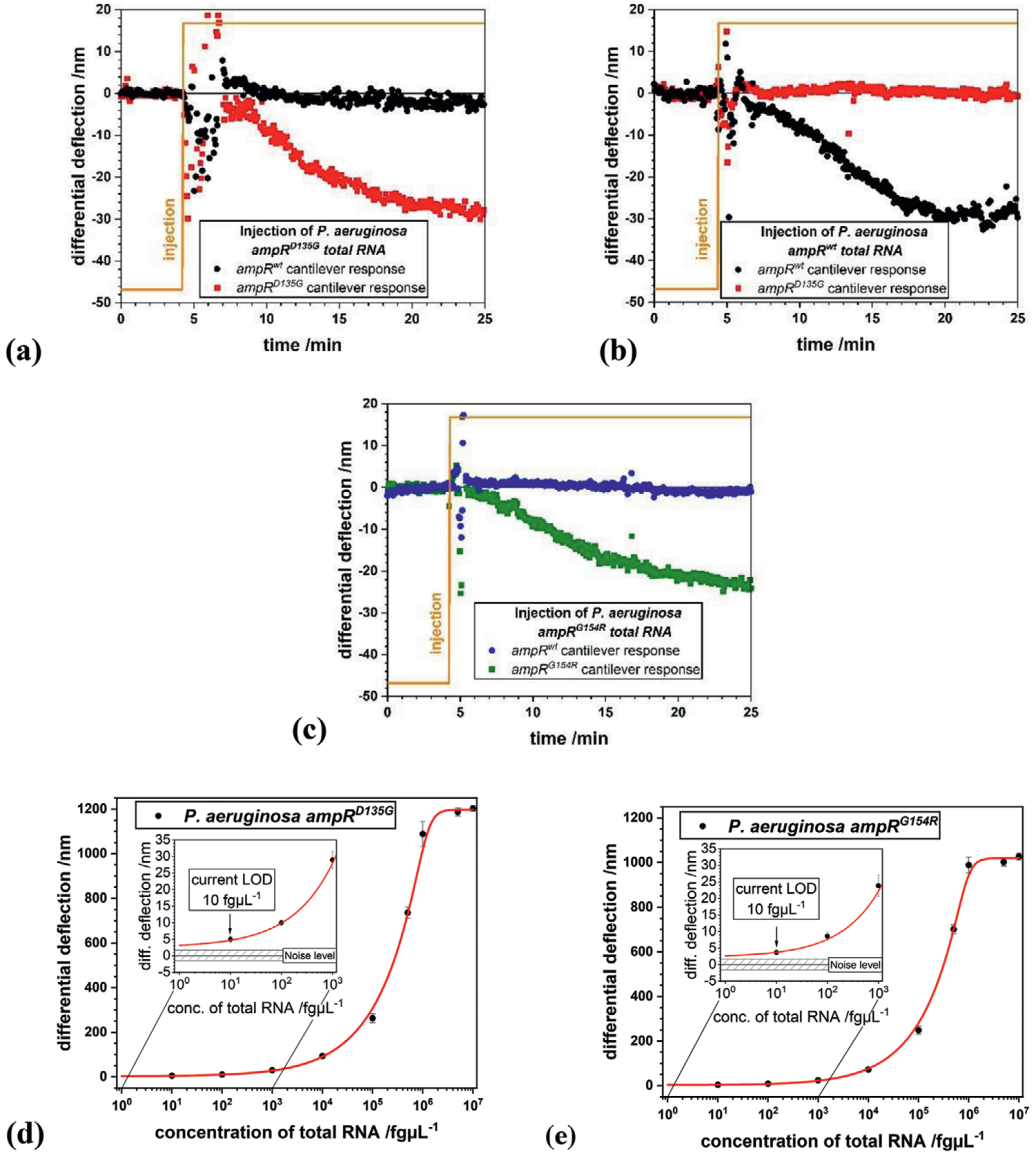
Differential responses to injection of 1 pg µL^−1^ total RNA. Differential measurements: a) Shown in red: difference of mutation D135G response and reference cantilever response. b) Shown in black: *P. aeruginosa* total RNA response from *ampR* wild type minus reference cantilever response. c) Shown in green: difference of mutation G154R response and reference cantilever response. Response magnitude is about 28 ± 3 nm at a concentration of 1 pg µL^−1^. Current limit of detection (LOD) for total RNA of *P. aeruginosa* d) *ampR^D135G^* and e) *ampR^G154R^* strains. The insets show the LOD of the measurement system of 10 fg µL^−1^ as determined from the logistic fit. Cross sensitivity is less than 2 nm.

Based on previous nanosensor experiments,^[^ ^22^ ^]^ we found that this position yields optimized results. The four oligonucleotides consisted of wild type, *ampR^D135G^*, *ampR^G154R^*, and a different nucleotide at the position of the mutation as a reference termed *ampR^ref^*. We were able to distinguish *ampR^wt^* (**Figure** **3**a), *ampR^D135G^* (Figure 3b), and *ampR^G154R^* (Figure 3c) using corresponding matching sequences within a few minutes. Multiple mutations are probed using the same array to discriminate two different mutations and the wild type, literally toggling between the different sequences.

We performed experiments at 28 °C at various concentrations of total RNA from *P. aeruginosa ampR^D135G^* and *ampR^G154R^* (Figure 3d,e) in the range from 10 fg µL^−1^ up to 10 ng µL^−1^ to investigate the limit of detection (LOD) for SNPs using a logistic fit function (Equation 1). We obtained average signals in between 5 ±1 nm up to 1202 ±10 nm. Single point mutation specificity was demonstrated in these experiments using arrays with cantilevers of 750 µm length and 500 nm thickness. 1 pg of total RNA was injected, corresponding to a sensitivity of detection of merely ten bacterial cells.

(1) $$\begin{array}{c} D\left(c\right)=D_{\min}+\frac{D_{\max}-D_{\min}}{{\left(1+{\left(\frac{c_{0}}{c}\right)}^{h}\right)}^{s}} \end{array}$$

*D*(*c*) is equal to the deflection at a particular concentration *c*, *D*_min_ corresponds to the minimum deflection and *D*_max_ to the maximum deflection. h corresponds to the Hill coefficient at *c*_0_ equal to half *D*_max_, *s* is a control factor_._

### 2.2 Detection of Bacterial Genes

Second, we approached specific detection of antibiotic resistance genes, e.g., coding for genes producing enzymes able to cleave antibiotics or resulting in reduced binding affinities of an antibiotic to its target.^[^ ^23^ ^]^ Three different and common vancomycin resistance genes^[^ ^24^, ^25^ ^]^ called *vanA*, *vanB*, and *vanD* were investigated. The three genes are distinguished by multiple mutations within the genes. We chose 19 to 21 long oligonucleotides (**Table** **2**) from the 3′ terminal end as probes with similar hybridization temperatures for the experiments. In these experiments, a polyAC sequence of 19 bases was used as a reference sequence. We detected *vanA*, *vanB*, and *vanD* using total RNA extracted from *Enterococcus faecium*.

**Table 2:** Showing sequences of the four different oligonucleotides used for vancomycin resistance experiments. Capitalized letters indicate the differences in the *vanB* and in the *vanD* gene compared to the *vanA* sequence, whereby polyAC serves as reference

Designation	Sequence	Position range	*T*_m_ ^a)^
*vanA*	tcacccctttaacgctaat	1014 to 1032	27.5 °C
*vanB*	tcacc **T** ctttaacgc **C** aat	1011 to 1029	28.0 °C
*vanD*	t **T** acctc **C** t **A** a **GT** g **AA** a **G** tcc	1012 to 1032	27.9 °C
polyAC	acacacacacaacacacac	–	–

^a)^ *T*_m_ stands for melting temperature

**Figure 4:**
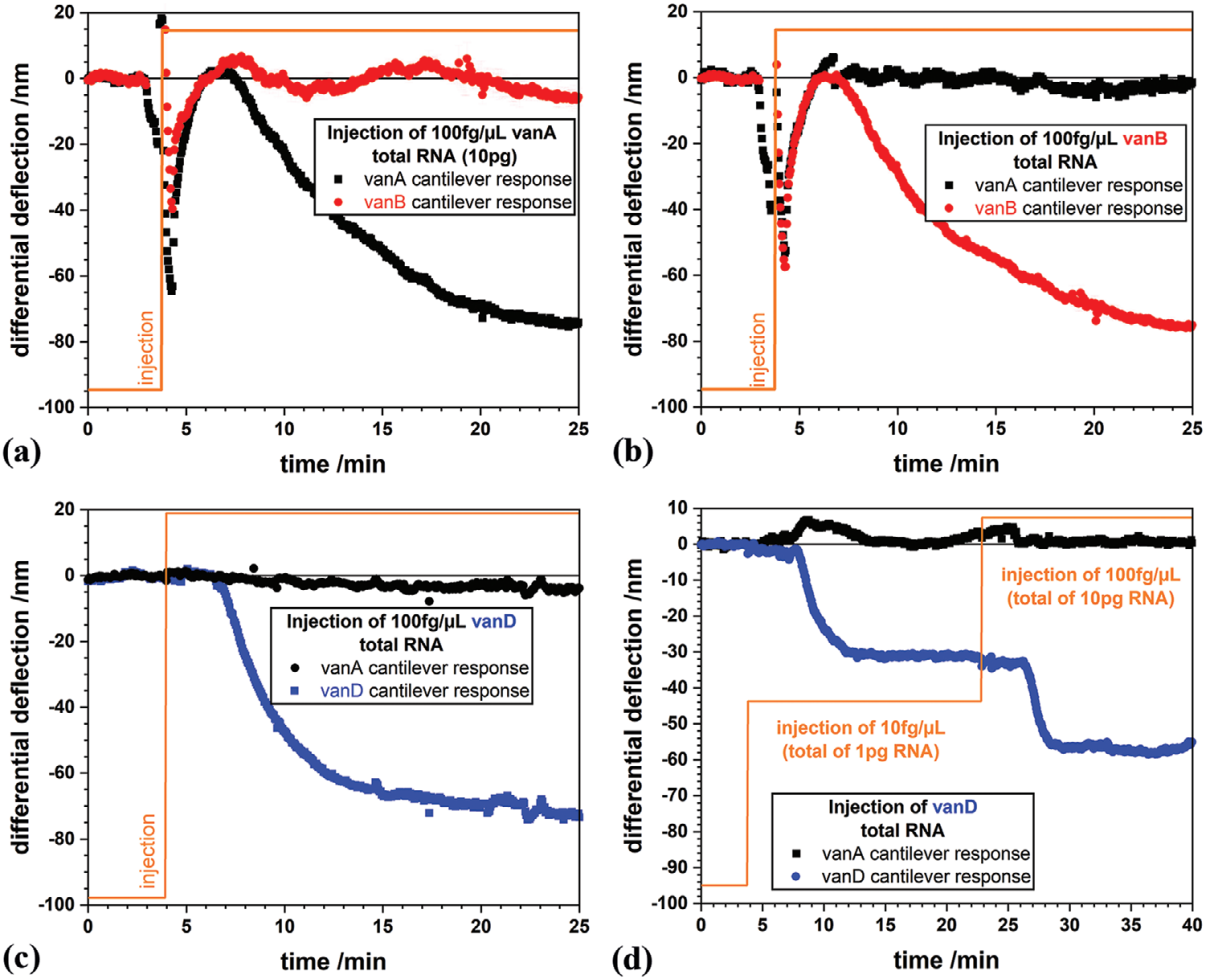
Specific differential responses to *vanA*, *vanB*, and *vanD* samples. A total of 10 pg RNA was injected in each case at a concentration of 100 fg µL^−1^. a) Response of *vanA* coated cantilevers upon injection of total RNA extracted from *vanA* producing *E. faecium* compared to *vanB* coated cantilevers. b) Response of *vanB* coated cantilevers upon injection of total RNA extracted from *vanB* producing *E. faecium* compared to *vanA* coated cantilevers. c) Response of *vanD* coated cantilevers upon injection of total RNA extracted from *vanD* producing *E. faecium* compared to *vanA* coated cantilevers. d) Sequential injection of RNA from *vanD* producing *E. faecium* at different concentrations. We started with a sample of 10 fg µL^−1^ at 20 min and changed to 100 fg µL^−1^ at 40 min.

We initially used RNA concentrations of 100 fg µL^−1^ for reliable and rapid detection of resistance genes (**Figure** **4**a–d) and injected in each case overall 10 pg total RNA, which is equivalent to approximately 100 bacterial cells.^[^ ^26^ ^]^ All three *E. faecium* RNA samples show a similar response of 80 nm indicating similar expression levels of the different vancomycin genes.

**Figure 5:**
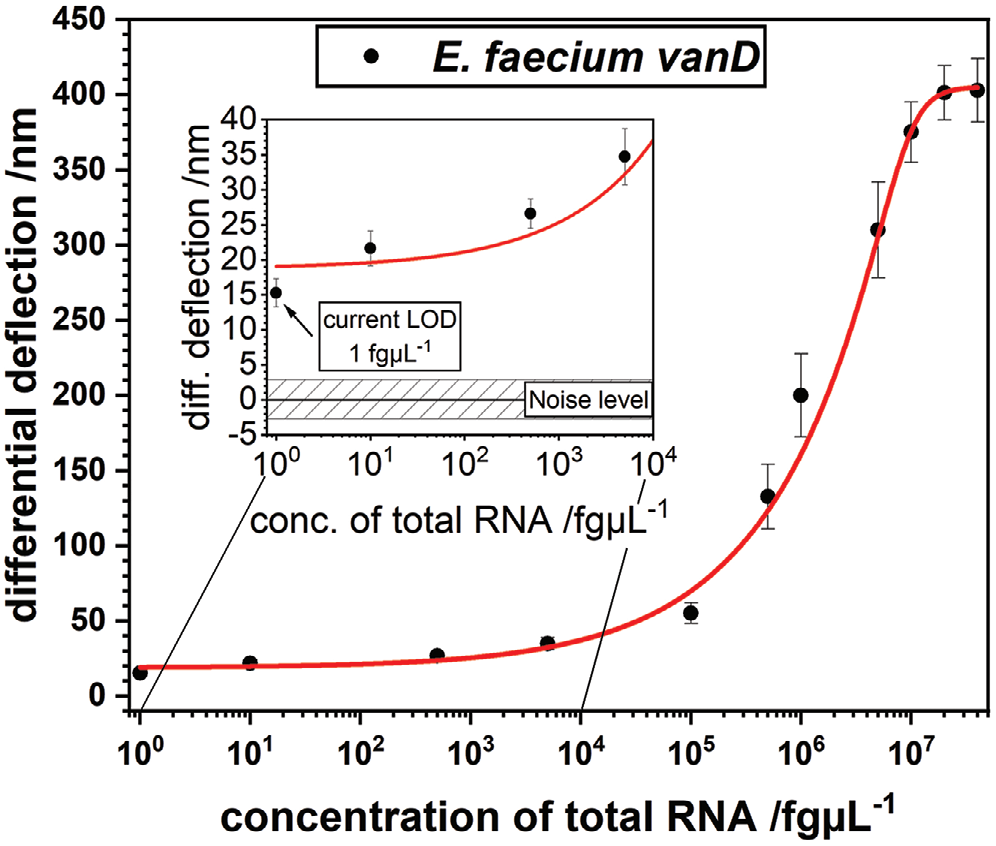
Establishing a limit of detection (LOD) for *E. faecium* total RNA of the *vanD* gene. Serial dilutions were measured from 1 fg µL^−1^ to 40 ng µL^−1^. A logistic fit was applied to determine the LOD at the minimum deflection *d*_min_.

A series of experiments with different concentrations in the range of 1 fg µL^−1^ to 40 ng µL^−1^ (**Figure** **5**) was conducted. **Table** **3** summarizes the corresponding cross reactivities between the probes on the sensors and the injected targets. The fact that cross reactivity signals from noncorresponding probes are only a few percent in magnitude of gene recognition signals from corresponding probes underlines the high specificity of this method.

**Table 3:** Cross‐reactivity matrix. The responses of the sensors functionalized with a specific probe to different target samples is shown. The corresponding signals are in bold. A ten to 20‐fold signal magnitude is observed compared to noncorresponding probes

	vanA probe	vanB probe	vanD probe
injection of vanA	**−75.3 ± 7.0 nm**	−5.6 ± 2.9 nm	2.3 ± 2.8 nm
injection of vanB	−2.9 ± 2.3 nm	**−75.4 ± 1.5 nm**	−4.4 ± 3.1 nm
Injection of vanD	−3.6 ± 3.7 nm	−1.3 ± 2.2 nm	**−55.0 ± 7.0 nm**

The upper limit in the differential deflection signal at 40 ng µL^−1^ is a result of the number of probes on the cantilever surface and the percentage that can bind to a target. These values were estimated to be 1.5 **×** 10^10^ oligonucleotides per nanosensor and a maximum of 10% have been shown to bind,^[^ ^27^ ^]^ respectively. 40 ng RNA will contain an estimated number of 1.6 **×** 10^9^ targets which corresponds to an occupancy of 10%. Therefore, as 10% of all possibly accessible probes are occupied, we almost reached the theoretical limit for binding.

**Figure 6:**
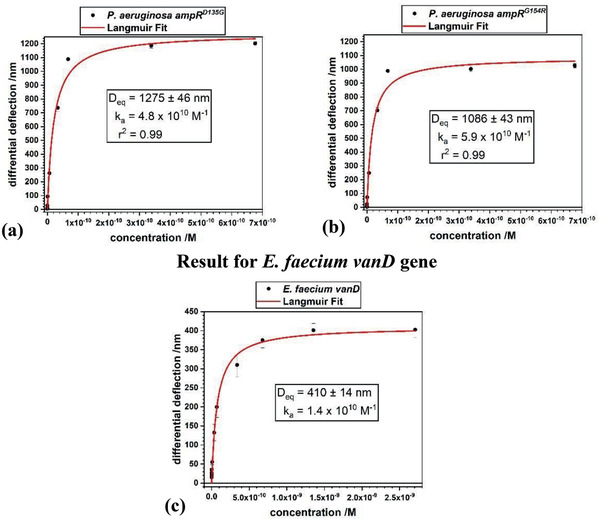
Langmuir analysis of data. The concentrations of *ampR*, a) *ampR^D135G^*, b) *ampR^G154R^*, between 10 fg µL^−1^ and 10 ng µL^−1^ as well as for *vanD* c) from 1 fg µL^−1^ to 40 ng µL^−1^ were converted into molar concentrations using an estimated mRNA content of 5% in total RNA and an average length of 1000 nucleotides with a molecular weight of 340 kD. The Langmuir isotherms with an *r* ^2^ = 0.99 and *r* ^2^ = 0.98, respectively, imply a reliable fit.

Determination of Gibbs free energy (**Figure** **6**) using Equation 2 for the hybridization of the *vanD* gene resulted in a value of Δ*G* = −58.38 kJ mol^−1^. The Gibbs free energy calculations gave a value of Δ*G*  = −61.5 kJ mol^−1^ for *ampR^D135G^* and −62.65 kJ mol^−1^ for *ampR^G154R^*. Values of Δ*G* for the point mutations are larger than for the whole gene detection. This finding justifies the observation of the higher equilibrium binding signal of around 1100 nm versus the 400 nm differential deflection signal for the *vanD* gene.

(2) $$\begin{array}{c} D\left(c\right)=D_{\text{eq}}\times \frac{k_{a}\times c}{1+k_{a}\times c} \end{array}$$

*D*(*c*) is equal to the deflection at a particular concentration *c*, and *D*_eq_ corresponds to the equilibrium deflection. The constant *k*_a_ is the hybridization equilibrium constant.

A total of 100 fg total RNA at the LOD (1 fg µL^−1^) was injected, which corresponds to a single bacterial cell. This finding demonstrates the ultrahigh sensitivity of our method, which will be beneficial in measurements from samples of body compartments with very low bacterial cell concentrations such as blood during sepsis or cerebrospinal fluid during meningitis.

## 3 Conclusion

In this study, we have explored representative antibiotic resistance mechanisms also commonly encountered in clinics—first, SNPs in the *ampR* gene modulating *ampC* gene expression were detected using probes that cover the location of the mutation, involving substantial overhangs up‐ and downstream embedding the mutation, since the polymorphism is usually located inside the gene and not at its ends. The overhangs reduce the signal due to steric hindrance, but do not compromise binding to the cantilever.

Second, genes associated with vancomycin resistance were targeted. For detection of a gene, we intentionally designed the probe location in such a way that it is at the end of the gene, thus further improving sensitivity due to the fact that nanomechanical responses are larger the closer they occur relative to a surface.^[^ ^28^ ^]^ In particular, we demonstrated detection of *vanD* RNA at only 1 bacterial cell per milliliter of fluid with high specificity.

A key advantage, in addition to the very high sensitivity and specificity, is the rapid response time of our system in the order of 5 min. The magnitude of the signals for the Langmuir plot and LOD analysis were obtained 20 min after the injection. In certain compartments of the patient only a few hundred bacterial cells per ml fluid may be present. A low detection limit generates the potential to diagnose bacteremia and meningitis directly from patient specimen. Our method presents a novel way to detect antibiotic resistance mechanisms at unprecedented speed and sensitivity. The technique is label‐free and allows nonamplified detection of resistance markers in total RNA from as little as 100 fg (corresponding to 1 bacterial cell) at a concentration of 1 fg µL^−1^ for vancomycin resistance and 1 pg (corresponding to ten bacterial cells) at a concentration of 10 fg µL^−1^ in SNP analysis of the *ampR* gene. These findings represent vast progress in rapid detection of antimicrobial resistance and open new avenues to investigate other infectious diseases or conditions where evolution of specific genes needs to be tracked at very low concentrations, e.g., sepsis, rare genetic variants. No significant influences were found so far in preliminary studies investigating bacterial resistance markers in blood based clinical samples to assess possible matrix background noise effects. This will show the feasibility of the method to investigate total bacterial RNA isolated from complex samples, such as blood.

## 4 Experimental Section

##### Clinical Isolates

The bacterial isolates (*Pseudomonas aeruginosa* and *Enterococcus faecium*) used were from the biobank of the division of Clinical Bacteriology and Mycology at the University Hospital Basel. Strains identification was confirmed using MALDI‐TOF mass‐spectrometry (Bruker). All strains were cultured on standard blood agar plates (5% sheep blood, bioMérieux) over night and a suspension with 0.5 McFarland was generated from which RNA was extracted using the RNeasy Mini Kit (Qiagen). Extracted RNA was quality controlled using Invitrogen Qubit 3.0 and Nanodrop 2000 (Thermo Fisher Scientific). Serial dilutions were prepared to determine the spectrum of sensitivity of the test system. All strains were sequenced using whole genome sequencing on a MiSeq (Illumina) platform at mean 30‐fold coverage and 2 **×** 300 nucleotide paired ends to confirm the molecular resistance mechanism.^[^ ^29^ ^]^ Sequencing information was used to determine specific probes for the SNP experiments. All genomes are available at NCBI GenBank (*P. aeruginosa* AE004091.2 and *E. faecium* AAAK03000003.1).

##### Nanosensor Array—Thiol Modified Oligonucleotide Probes

Thiol‐modified Oligonucleotide probes were ordered from Microsynth AG (Balgach, Switzerland) at a concentration of 100 × 10^−6^ m without Dithiothreitol (DTT). Prior to functionalization oligonucleotides were diluted to a concentration of 40 × 10^−6^ m in HPLC grade water containing 50 × 10^−3^ m tri‐ethyl‐ammonium‐acetate (TEAA) buffer and 1 × 10^−3^ m tris(2‐carboxyethyl)phosphine (TCEP). TCEP was used to reduce disulphide bonds of thiolated oligonucleotide probes. Chemicals and buffers were purchased from Sigma‐Aldrich.

##### Nanosensor Array—Nanosensor Array Preparation

Thinner and longer cantilevers yield higher sensitivity representing a major technical advantage compared to previous sensors. Before Au coating, the arrays were cleaned in Piranha solution (30% H_2_O_2_:96% H_2_SO_4_ = 1:1 v/v) for 10 min, rinsed three times with water followed by ethanol and dried in air. The upper sides of cantilevers were coated with a 3 nm‐layer of Ti followed by a 20 nm thick Au layer. While extracting DNA/RNA, nanosensor arrays were functionalized with thiol‐modified oligonucleotides at a concentration of 40 × 10^−6^ m using a modified ink‐jet spotting system (MDP705L, Microdrop Technologies, Norderstedt, Germany).^[^ ^30^ ^]^ Upon dispensing and subsequent incubation for an hour at room temperature, the array was mounted in the measurement chamber containing 0.03 **×** SSC (saline sodium citrate buffer, prepared using 20 x SSC from Sigma Aldrich). Arrays can be functionalized individually (typically three specific probe sensors, two wild type thiol oligonucleotide probes and three specific reference probes). It is of vital importance that differential measurements are performed. External factors such as nonspecific interactions and thermal drift are eliminated by calculating differential responses of probe and reference sensors (in this study derivatized with a nonspecific oligonucleotide of the same length as the probe sequence or, in some experiments, with a wild‐type sequence).

Total RNA was dissolved and serial dilutions were carried out all in DEPC treated water (0.1% diethylpyrocarbonate). For the experiments total RNA was finally diluted in 0.03 **×** SSC. All measurements were performed on a home‐built sensor instrument. The bending of cantilevers was detected by reflection of an external laser beam focused at the cantilever apex. The instrument enables monitoring the deflection of all eight sensors in parallel in a time‐multiplexed manner. All measurements were performed at 28 °C in a temperature‐controlled box with a steady buffer flow of 5 µL min^−1^, which was found optimal for this type of experiment. Data acquisition hardware, temperature regulation, and a syringe pump for buffer and sample injection were controlled by dedicated LabView software.

## Conflict of Interest

The authors declare no conflict of interest.

## Author Contributions

D.L. prepared the bacterial isolates and extracted the RNA. V.H. characterized the samples. D.W. analyzed the whole genomes. F.H., H.P.L., C.G., E.M., and A.E. conceived and designed the experiments. F.H. and H.P.L. prepared the nanosensor arrays. F.H. functionalized the sensors, performed the experiments and analyzed the data. F.H., H.P.L., C.G., E.M., and A.E. wrote the manuscript, critically advised and contributed in interpreting the results. A.E., E.M., and C.G. were in charge of the overall direction and planning of this study, and all the authors commented on the manuscript.
